# The Mediating Role of Loneliness in the Association Between Oral Health and Mental Health in Older Adults

**DOI:** 10.1192/j.eurpsy.2025.1761

**Published:** 2025-08-26

**Authors:** C. Tsironis, M. Mantzoukas, A. Nakou, E. Bartzou, E. Dragioti, M. Gouva

**Affiliations:** 1 Research Laboratory Psychology of Patients Families and Health Professionals; 2 Nursing, University of Ioannina, Ioannina, Greece

## Abstract

**Introduction:**

Loneliness is widely recognized as a significant risk factor for psychosomatic issues in older adults, potentially impacting various aspects of health, including oral health.

**Objectives:**

This study aims to investigate the relationship between loneliness and oral health in older adults.

**Methods:**

This cross-sectional study was conducted with a sample of 84 older adults (41 females and 42 males), aged between 65 and 94 years (mean age: 74.1 years, SD = 8.1). Participants completed a sociodemographic questionnaire, the 12-item Geriatric Oral Health Assessment Index (GOHAI), and the Emotional and Social Loneliness Scale. Multivariate analysis was used to assess the impact of loneliness on oral health outcomes.

**Results:**

The analysis revealed that emotional and social loneliness had a significant negative impact on oral health. Furthermore, the overall loneliness score was strongly associated with poorer oral health quality, independent of marital status or the presence of children. In other words, the relationship between loneliness and oral health was not moderated by these demographic factors.

**Conclusions:**

As loneliness increases in older adults, their susceptibility to poor oral health rises, which can have significant implications for their psychological well-being. This study underscores the need to consider oral health as an integral component of overall well-being, particularly in the context of mental health in older populations.

**Disclosure of Interest:**

None Declared

